# Interferon-α induced remission in three patients with eosinophilic granulomatosis and polyangiitis. A case study

**DOI:** 10.1016/j.rmcr.2013.09.004

**Published:** 2013-09-26

**Authors:** B. Seeliger, M. Foerster, T. Neumann, A. Moeser, J. Happe, N. Kehler, C. Kroegel

**Affiliations:** a Pneumology & Allergology/Immunology, Internal Medicine I, Jena University Hospital, Germany; b Rheumatology/Osteology, Internal Medicine III, Jena University Hospital, Germany

**Keywords:** Eosinophils, Interferon, Vasculitis, Autoimmunity, EGPA, eosinophilic granulomatosis with polyangiitis, IFN-α, interferon-alpha, ANCA, antineutrophil cytoplasmatic antibodies, PNP, polyneuropathy, BAL, bronchoalveolar lavage, MU, Million Units, DMARDs, disease modifying anti-rheumatic drugs

## Abstract

Eosinophilic granulomatosis with polyangiitis (EGPA) is a systemic small vessel vasculitis associated with asthma and eosinophilia. Optimal therapy for maintenance of remission is yet to be defined. We present a case-series of three patients with EGPA in whom IFN-α, an immunomodulatory cytokine induced remission, which was maintained even after discontinuation of the drug. In all patients (ages 60, 51, and 50 years), remission was associated with normalisation of eosinophil counts and IgE-levels. Moreover, the patients remained in remission for one to four years. Two patients did not need further immunosuppression, one patient required low dose maintenance therapy.

Although reversible side effects occur, IFN-α-therapy induces long-term remission of EGPA even after discontinuation of treatment.

## 1 Introduction

EGPA is a rare systemic necrotizing vasculitis of the small-vessels, first described as allergic granulomatosis and angiitis in 1951 [1]. It is characterized by bronchial asthma with pulmonary infiltrates, peripheral eosinophilia and involvement of various organs such as heart, peripheral nerves, kidneys and the gastrointestinal tract. EGPA has been categorized as antineutrophil cytoplasmatic antibodies-associated-vasculitis, however only 30–40% of the patients are ANCA positive [2], [3].

There exist several treatment options for induction of remission, but relapses are frequent and the most effective and safest therapy for maintenance of remission is yet to be defined.

IFN-α-therapy inhibits eosinophil degranulation and potentially reverses TH2-mediated immune responses [4]. Several case series indicate its efficacy [5], [6], [7] in treating EGPA and a recent prospective clinical trial [8], [9] demonstrated that IFN induces remission. Here, we report the course of three ANCA-negative patients [Table 1] with severe EGPA treated with IFN-α for up to 131 months.

**Table 1:** Characteristics of 3 ANCA-neg. patients with EGPA treated with IFN-α (Part 1).

Pat.	Age, sex	FFS	ANCA	DEI	Disease duration [months]^a^	Previous treatment	PRD [mg/d]	Histology^b^	Clinical features
1	60, f	0	Neg.	6	36	PRD	Irr. 100 mg pulses	L	Arrhythmia, eosinophilic alveolitits, mononeuritis multiplex
2	61, m	0	Neg.	6	61	PRD, iv.CYC	0^c^	–	Mononeuritis multiplex, eosinophilic alveolitis
3	50, f	0	Neg.	4	120	PRD	100	–	Eosinophilic alveolitis

FFS: Five-factor score, DEI: disease extent index, PRD: prednisolon, i.v.CYC: intraveneous cyclophosphamide, L: lung.

a Prior to IFN-α-treatment.

b Biopsy proven vasculitis.

c PRD-dosage was tapered and discontinued two weeks prior to treatment from 70 mg/d.

## 2 Case reports

### 2.1 Case 1

A 60-year-old female non-smoker presented with progressive dyspnoea and airway obstruction in 1999. Past medical history included allergic diathesis, chronic sinusitis and refractory tachycardia. The patient repeatedly received i.v. corticosteroids. Polyneuropathy (PNP) of both legs occurred one year before admission. X-rays of the chest over the past two years showed migrating infiltrates. Past lab-exams revealed peripheral eosinophilia of 38%. ANCA antibodies could not be detected. Bronchoscopy and bronchoalveolar lavage (BAL) showed significant eosinophilia of 61.5%. Transbronchial mucosal biopsies revealed eosinophil infiltrations and eosinophil vasculitis. Thus, the patient met all six ACR diagnostic criteria for EGPA.

At first presentation, the patient scored a BVAS of 21. Treatment with IFN-α2b (9 Million Units (MU) per week) was initiated. Under therapy, the patient showed remarkable clinical improvement and remission was induced after 6 months (BVAS = 0). The area of sensoric neurologic deficit of the leg regressed 20 cm distally following 24 months of treatment. Her initial blood eosinophil count decreased rapidly [Table 2]. Except for discrete hyperinflation, pulmonary function tests normalized after 12 months of treatment.

**Table 2:** Characteristics of 3 ANCA-neg. patients with EGPA treated with IFN-α (Part 2).

Pat.	Before therapy			Remission			Relapse		
	Eos [10^9^/l]	IgE [IU/ml]	FEV_1_ [l]	Eos [10^9^/l]	IgE [IU/ml]	FEV_1_ [l]	Eos [10^9^/l]	IgE [IU/ml]	FEV_1_ [l]
1	896 (14%)	859	2.23 (83%)	45 (1%)	–	2.57 (103%)	118 (2%)	466	1.62 (61%)
2	720 (10%)	509	2.57 (79%)	84 (3%)	1170	2.72 (85%)	–	–	–
3	1080 (12%)	190	1.27 (45%)	430 (10%)	194	2.57 (92%)	1386 (21%)	–	2.32 (83%)

**Fig. 1:**
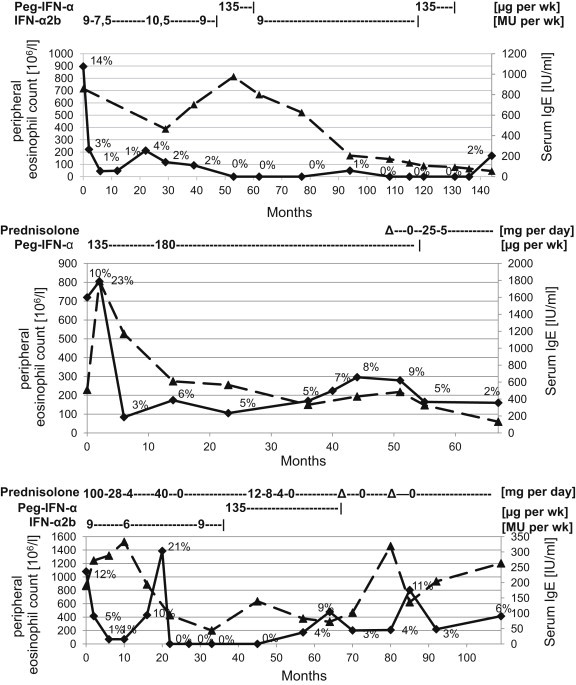
**Eosinophil count** (solid line) in absolute numbers and percentage of all leukocytes and **total serum IgE** (dashed line) concentration and IFN-α2b, Peg-IFN-α and prednisolone dosages before, during and following treatment with IFN-α of all three patients with the EGPA. Δ indicates short-term PRD therapy starting at 40 mg/d due to infection. Case 1. **Upper**. Case 2. **Middle**. Case 3. **Bottom**. MU = Million Units, IFN-α = interferon-α, Peg-IFN- = pegylated Interferon-α.

The patient remained asymptomatic until she suffered a minor relapse following 26 months of therapy (BVAS = 6) [Table 2]. Within a total of 131 months of IFN-therapy, IFN dosages and preparation was adjusted as shown in Fig. 1. Nine years after initiation of therapy, PNP progressed. As asthma and sinusitis remained clinically stable and peripheral eosinophil count was at 53 × 10^9^/l (1%), IFN-induced neuropathy was suspected, which remained after switching treatment to PEG-IFN-α. Therefore, IFN-α was discontinued, which was followed by clinical improvement of neuropathy. After 12 months PNP had not progressed, and serum IgE-level fell to 55.1 IU/ml with an eosinophil count of 170 × 10^9^/l (2%) and no ENT or asthma-symptoms. The patient remained in complete remission for 12 months without IFN or systemic prednisolone [Table 3].

**Table 3:** Outcome of 3 patients treated for remission and maintenance with IFN-α.

Pat.	IFN-therapy [Months]	Follow-up [Months]	Side effects [Management]	Relapses (Month) [Management]	Outcome
1	131	144	Progress of PNP [discontinuation of IFN-α]	Asthma exacerbation (26) [IFN-α↑]	IFN-α discontinued (remission without immunosuppresive agents)
2	55	67	Autoimmune hepatitis [discontinuation of IFN-α]	None	IFN-α discontinued, recurrence of asthma
3	61	109	Hyperthyreosis, anaemia [discontinuation of IFN-α]	Mononeuritis multiplex (20) [IFN-α↑, PRD↑]	IFN-α discontinued (remission without immunosuppresive agents)

↑ = increase in the dosage, ↓ = decrease in the dosage.

### 2.2 Case 2

A 60-year-old male non-smoker presented in 2003 with a history of asthma, chronic sinusitis with anosmia and mononeuritis multiplex of both hands and feet as well as atrophy of the left forearm muscles. Laboratory exams revealed a peripheral blood eosinophil count of 650 × 10^9^/l (10%) and a serum IgE-level of 599 IU/ml. Bronchoscopy and BAL showed significant eosinophilia of 15%. Meeting four of six ACR diagnostic criteria, the patient was diagnosed with EGPA.

The patient received oral corticosteroids for 7 months and subsequently 11 pulses of cyclophosphamide over a period of 17 months. However symptoms relapsed and IFN-treatment was initiated [Table 2].

Following two months of IFN-therapy, the patient reported complete regression of dyspnoea and anosmia with improved lung-function tests, which was followed by no further progression of PNP. After 6 months, complete remission was induced (BVAS = 0). Side effects including arthralgia and muscle pain after IFN-injection were transitory.

Following 55 months of treatment, IFN-therapy was discontinued due to development of an ANA+ and SMA+ autoimmune hepatitis. Prednisolone therapy (25 mg/d) was initiated, leading to normalization of liver enzymes. Prednisolone was tapered to 5 mg as a maintenance therapy within three months. After discontinuation, asthmatic complaints recurred.

### 2.3 Case 3

A 50-year-old female non-smoker presented with dyspnoea, sinusitis and airway obstruction. The patient had a history of asthma and polyvalent allergy followed by chronic sinusitis. Eight years prior to admission, symptomatic prednisolone therapy (10–100 mg/d) was initiated due to severe dyspnoea.

On admission, the laboratory exams and pulmonary function tests showed an elevated peripheral eosinophil count and a severe airway obstruction [Table 2]. BAL revealed a significant bronchoalveolar eosinophilia. An X-ray of the chest showed pulmonary infiltrates. At the time of presentation, the patient scored a BVAS of 8 and the diagnosis of EGPA was established.

In order to induce remission, 9 MU of IFN-α2b per week were administered. Under therapy, the patient showed significant clinical improvement. Within two months of IFN-α, the prednisolone dosage could be decreased to 4 mg/d. Side effects are listed in Table 2. The peripheral eosinophil count dropped to 415 × 10^9^/l (5%) after two months and was at 69 × 10^9^/l (1%) after five months of therapy. Additionally, the FEV_1_ improved from 1.27 L (44%) to 2.17 L (77%) after two months of IFN-α therapy.

Following six months of therapy, prednisolone could be discontinued. A subsequent respiratory infection resulted in a temporary re-administration of prednisolone, which could be tapered and discontinued three months later. At that time, after 16 months of treatment complete remission was induced (BVAS = 0). Following twenty months of therapy, the patient suffered a relapse (BVAS = 11) and presented with worsening of PNP and elevated peripheral eosinophil count [Table 2]. IFN-α-dosage was increased, combined with prednisolone starting with 40 mg/d. IFN-α was switched to Peg-IFN-α due to enduring fatigue and complete remission was achieved. Following two months of Peg-IFN-α, the peripheral eosinophil count dropped to 0% and the serum IgE-level decreased from 93.7 IU/l to 43.8 IU/l one year after administration of Peg-IFN-α. Prednisolone was tapered and could be discontinued 18 months after relapse without recurrence of symptoms.

After five years, IFN-α-therapy was discontinued due to slowly progressive myelosuppression (erythrocyte count of 2.9/pl). Since then the patient remained in remission without prednisolone.

## 3 Discussion

The conclusions of the case series presented herein are threefold. Firstly, the cases confirm previous observations [5], [6], [7], [8], [10] showing that IFN induces complete remission in patients with EGPA. Secondly, the study extends previously published data and demonstrates that remission is maintained under treatment for up to ten years. Thirdly, the case reports demonstrate for the first time that remission is maintained up to four years after IFN therapy has been discontinued. Because IFN inhibits the Th2 immune pattern [11], the data suggest that the cytokine shows a long-lasting immunomodulatory action in EGPA, which persist even after treatment has been terminated.

In EGPA, treatment goals in the past mainly focused on symptom relief and disease control whilst little attention has been paid to long-term remission or even cure as an achievable therapeutic goal. Corticosteroids alone or in combination with immunosuppressants are the mainstay of therapy and usually improved symptoms and reduced the frequency of severe exacerbations. However, treatment is limited by poor efficacy or toxicity and relapses are likely with low dosages or discontinuation. In addition, both spontaneous remission and treatment-induced long-term maintenance of remission are uncommon.

With the introduction of disease-modifying drugs and biologics, which, in contrast to standard immunosuppressive drugs, selectively intercept one specific disease pathway, remission has become a realistic treatment goal. For instance, in rheumatoid arthritis (RA), another autoimmune disease, blocking the activity of tumour necrosis factor (TNF) using anti-TNF-antibodies over a period of four years leads to remission of RA in 43% [12] and in 13% of these patients drug therapy can even be discontinued for a prolonged period [13]. The observations made in RA may have interesting parallels with EGPA. Both RA and EGPA are autoimmune disorders and both are treated with standard immunosuppressive treatment including corticosteroids and immunosuppressants. In both diseases spontaneous and treatment-induced remission using standard immunosuppression regimens are uncommon. Hence, in contrast to standard treatment, the case series presented herein suggest that immunomodulatory treatment of EGPA with IFN induces remission that may continue for several years. In fact, immunomodulatory therapy may be superior to standard immunosuppression as it may induce long-term remission even after discontinuation of treatment.

Little is known regarding maintenance of remission in EGPA treated with IFN. A small study with 13 patients reported a mean time to first relapse of 17 months [9]. In RA, almost half of the patients in whom maintained remission was achieved after discontinuation of therapy with DMARDs experienced a relapse during a 15-year follow-up period [14]. Thus, we cannot exclude that some, if not all, patients with EGPA in remission presented herein may also develop a relapse in the future.

In RA, a proportion of patients with apparent clinical remission showed signs of progressive joint damage indicating subclinical disease activity [15]. This may be similar to the cases presented above, which showed an increase in IgE serum levels and peripheral eosinophil counts whilst still being in clinical remission. In addition, there was no indication for organ involvement including heart and lung. However, whether these biological parameters precede a relapse in EGPA remains unclear.

IFN-treatment may cause significant adverse effects. All three patients experienced early side effects like arthralgia, myalgia and malaise and patient 3 also experienced depression after IFN-injections, but those side effects were transient. Patient 3 also developed hyperthyroidism, but was euthyroid under methimazole. Eventually, all patients had to discontinue IFN due to adverse events that disappeared shortly after discontinuation of treatment or did not progress further (PNP of patient 1).

Recent studies have shown that rituximab, a B-cell-depleting anti-CD20 monoclonal antibody and mepolizumab, an anti-IL-5-antibody also induce remission in EGPA [16], [17], [18]. However, data on long-term efficacy in maintaining remission after discontinuation of treatment are not available making a reliable comparison of IFN-α to rituximab or mepolizumab impossible.

We fully acknowledge the limitations of an observational study in only three patients. For instance, the patients presented were ANCA-negative. Thus, the beneficial effect of IFN may not apply to ANCA-positive cases. Previous reports [7], [8] however have demonstrated that ANCA-positive patients equally respond to IFN-α. We also are aware that IFN may cause side effects. However, these adverse reactions were reversible. In addition, we believe that this study provides important preliminary evidence that IFN may be effective in inducing and maintaining remission in severe EGPA making it superior to standard immunosuppression which is often limited by poor efficacy or toxicity. Obviously, randomized, double-blind and controlled trials need to be conducted to assess whether IFN is preferable in achieving long-term maintenance of remission in patients with severe EGPA.

## Conflict of interest statement

Authors confirm that there are no known conflicts of interest associated with this publication and there has been no significant financial support for this work that could have influenced its outcome.
